# *Mindful Melody:* feasibility of implementing music listening on an inpatient psychiatric unit and its relation to the use of as needed medications for acute agitation

**DOI:** 10.1186/s12888-021-03127-z

**Published:** 2021-03-06

**Authors:** Trevor Scudamore, Annette Liem, Mark Wiener, Nekpen Sharon Ekure, Christopher Botash, Derek Empey, Luba Leontieva

**Affiliations:** SUNY Upstate Medical University, 750 East Adams Street, Syracuse, NY 13210 USA

**Keywords:** Agitation, Inpatient psychiatric unit, Music, As needed medications (PRN), Coping skills, Restraints, Seclusions

## Abstract

**Background:**

In this quality improvement project, we set out to study the effectiveness and feasibility of using music as an adjunct or replacement for pharmacologic agitation management on an inpatient psychiatric unit. We hypothesized music intervention would not only assist in de-escalation/calming of agitated patients, but also reduce overall administration of PRN medications on the unit.

**Method:**

The project included 172 volunteer participants over 6 months: Three months without music available and 3 months with a music de-escalation option. During the latter period, patients were given the option of selecting a preferred music genre and provided with wireless headphones for up to 30 min. The number of as needed (PRN) medications administered for agitation and anxiety (including oral, sublingual, and intramuscular routes) was compiled from raw data using pharmacy records. Patients and nurses were provided with self-report surveys regarding the music intervention.

**Results:**

The average weekly PRN medication administrations decreased significantly during the 3 months with music for both haloperidol (8.46 [+/− 1.79, *p* < 0.05] to 5.00 [+/− 1.44, *p* < 0.05] administrations/week) and olanzapine (9.69 [+/− 2.32, *p* < 0.05] to 4.62 [+/− 1.51, *p* < 0.05] administrations/week), compared to the 3 months prior to music implementation. There was a non-significant increase in administration of lorazepam (3.23 [+/− 1.09, *p* < 0.05] to 6.38 [+/− 2.46, *p* < 0.05] average administrations/week). The patient survey responses were 96% positive (non-neutral; either agree or strongly agree with calming effect). Nurses agreed that the project was easy to implement; 56% agreed that music helped to calm patients down. Other exploratory outcomes included observed reductions in average length of hospital stay and number of seclusion events.

**Conclusion:**

Music may play a significant role in reducing the utilization of PRN agitation medications on acute inpatient psychiatric units. More studies are needed to expand on these findings and explore the effect of PRN music on other therapeutic outcomes.

**Trial registration:**

Protocol registration NCT04514432, retrospectively registered on 08/13/2020.

**Supplementary Information:**

The online version contains supplementary material available at 10.1186/s12888-021-03127-z.

## Background

Agitation and anxiety are frequent events on inpatient psychiatric units. These occurrences put stress on both health care providers and patients, potentially compromising the therapeutic milieu and extending hospital stay [1]. Agitation is commonly treated with medication; usually an antipsychotic such as haloperidol or olanzapine, or an anxiolytic such as lorazepam or hydroxyzine. These medications may be given orally or, if refused and deemed necessary, intramuscularly. While this pharmacologic intervention may temporarily diffuse the situation, there are costs: monetarily, to the patient relationship, and the patient’s own health [2]. This paper evaluates listening to music as a potentially safe and effective alternative for treating agitation. As a disclaimer, music therapy as defined by the American Music Therapy Association is “the clinical and evidence-based use of music interventions to accomplish individualized goals within a therapeutic relationship by a credentialed professional who has completed an approved music therapy program” [3]. Therefore, this project does not meet “music therapy standards,” but instead is an alternative for providers and units who do not have access to credentialed music therapists. For the purposes of this project, “music listening” will be used to describe the offer of music to patients.

According to Dr. Zeller of Project BETA (Best Practices in Evaluation and Treatment of Agitation), agitation is defined as “excessive verbal and/or motor behavior that can be loud, disruptive, hostile, sarcastic, threatening, hyperactive, and/or combative” [4]. Agitation is more than just an aspect of the psychiatric disease process, but also a reaction to unmet psychosocial needs and attempts to communicate those needs. Other contributing factors may play a role when agitation escalates to the point of violent behavior. Such factors include lack of staff, increased staff frustration, training deficits, and small unit size [5, 6].

Multiple clinical studies present evidence that music listening may be beneficial in improving mood and treating a variety of symptoms (e.g., agitation, insomnia, and anxiety) in cases of depression, autism, schizophrenia, and dementia [7–10]. Patients may choose from numerous genres and artists, and music can be a cost-effective, low-risk method to augment a patient’s treatment (Marwick, 2000).

In a study by [11] on an acute inpatient unit, music was the most frequently used independent therapeutic nursing intervention to decrease agitation, anxiety, aggression, and depression, as well as to improve sleep. Other interventions included deep breathing, therapeutic touch, and massage, but music was most widely used on psychiatric and intensive care units [12]. presented an investigative model that includes attention, emotion, cognition, behavior, and communication modulation. Musical experiences and the relationships that develop through them can be potent conduits for change. For this reason, use of pre-recorded music to ease behavioral and psychological symptoms is popular in health-care contexts: both as formal music therapy sessions and in non-therapist-led interventions.

Though a combination of lyrics, melody and beat, music may form a calming stimulus, serving as a therapeutic intervention for agitated patients. A genre that is therapeutic for one patient may not be for another. As an example, classical music might elicit negative memories or induce emotions such as sadness or anger [13] and might have the opposite effect for others. To find the most effective music, patients at their baseline should be provided samples of different types of music genres, to see which they find the most relaxing [14].

The Cochrane Review for Schizophrenia studied the effects of music therapy alone, compared to music therapy in conjunction with standard care, standard care alone, placebo therapy, or no treatment for serious psychiatric illnesses. It concluded that music therapy, used in combination with standard care on a short- or medium-term basis, was helpful for patients with schizophrenia, benefiting their social functioning, global/mental state, and quality of life [15].

[16] suggest music may play an adjunctive role in supplementing medications for mildly to moderately agitated patients. In addition, music allows patients to practice self-soothing and fosters a sense of agency by decreasing reliance on as-needed (PRN) medications. Use of PRNs for agitation, while helpful in certain severe cases where patients are in danger of harming themselves or others, may be unnecessary if the patient is only mildly agitated. In these mild cases, medications are sometimes administered out of convenience, when ordering providers are not able to assess the patient face-to-face. Patients who have personality disorders, such as borderline personality disorder, may intentionally become agitated to receive intramuscular injections [17, 18]. Furthermore, all medications have potential side effects that may be permanent or deadly; extrapyramidal symptoms (EPS), neuroleptic malignant syndrome, QTc prolongation, cardiac arrhythmias, and allergic reactions are all possible risks, especially if given to drug-naïve patients.

In one review of the neural effects of music, emotion regulation is described as one of the methods people may use to remain comfortable in an anxious or agitated state [19]. Music is thought to affect many parts of the brain, including the amygdala, known for integrating emotions, the orbitofrontal cortex (OFC), and anterior cingulate cortex (ACC). The OFC and ACC, in communication with the amygdala, may have roles in modulating agitation [20]. Moore found that listening to familiar or preferred music affected the amygdala, OFC, and ACC positively, which helped enable emotion regulation.

We designed a quality improvement project to assess the feasibility and effectiveness of music listening as an alternative to PRN medication administration on an acute inpatient psychiatric unit. We hypothesized music listening would not only assist in the de-escalation and calming of mildly agitated patients, but also reduce overall administration of PRN medications. Furthermore, we wanted to assess whether a calmer therapeutic milieu was perceived by unit staff.

## Methods

Our quality improvement project was conducted exclusively on an acute 24-bed adult inpatient psychiatric unit at SUNY Upstate University Hospital, located in Syracuse, New York. The unit admits patients from the emergency department, medical floors, and surrounding facilities. Patients were provided with the option of listening to music as an alternative intervention to receiving medications (e.g., anxiolytics/sedatives, antipsychotics) for agitation, when observed by nursing staff. In some cases, patients endorsed agitation to nursing staff and self-requested music for de-escalation. On admission, all patients were made aware of the music de-escalation option and provided with information on the project; 172 patients were included in the study. Music was available for patients from 8 am until 10 pm outside of sleep hours. The total duration of the project was 6 months, from May 18, 2018 through November 13, 2018. The project included two groups: 3 months of baseline conditions without music available and 3 months with music available for de-escalation. PRNs administered for agitation and anxiety (including oral, sublingual, and intramuscular routes) were compiled from raw data using pharmacy records. Both pharmacy records and individual patient charts were reviewed to ensure accurate indication for PRN medication administration.

Patients were given the option of selecting a preferred music genre. For ease of implementation and to reduce the burden placed on nursing staff, only the genre playlists automatically generated by Spotify were offered. The most common genres included Hip Hop, Jazz, Blues, Rock and Pop. Nursing staff and patients could not select specific songs. If patients did not like a song in the queue, they were able to skip the song with Bluetooth headphone controls. This patient-centered approach, prioritizing patient involvement in his or her own treatment, has been shown to be of therapeutic benefit [16, 21]. To reduce the risk of a negative emotional response, when making their music selection, patients were also encouraged to alert staff of any song or genre that could potentially be triggering or offensive. Nursing staff would then choose an appropriate playlist and provide flexible short-stem Bluetooth headphones (with low potential for self-injury or harm), which were pre-approved by the SUNY Upstate Institutional Review Board (IRB). A training meeting was held with nursing staff to convey the purpose of the quality improvement project. Nurses were provided with a written protocol for administering headphones when requested, as well as instructions on how to operate and maintain the various electronic devices involved in the project. The headphones were collected after 30 min of listening, unless returned prior to completion of the session. During listening, patients were free to roam the unit or sit in an open area, but they were discouraged from isolating themselves in their rooms due to ligature and swallow risk, as they could not be monitored by staff. Patients were not allowed to share headphones.

Medications remained available after the listening period and patients were then asked to complete a brief self-report Likert scale survey to assess the degree of calming effect (Additional file 1).

The purchases needed to conduct this project included ten Bluetooth headphones, an iPad, and a three-month subscription to Spotify music streaming service. The costs were $20 each, $140, and $10 monthly, respectively, before tax. The total expense of completing this project is estimated to be $370.

### Data analysis

**Fig. 1:**
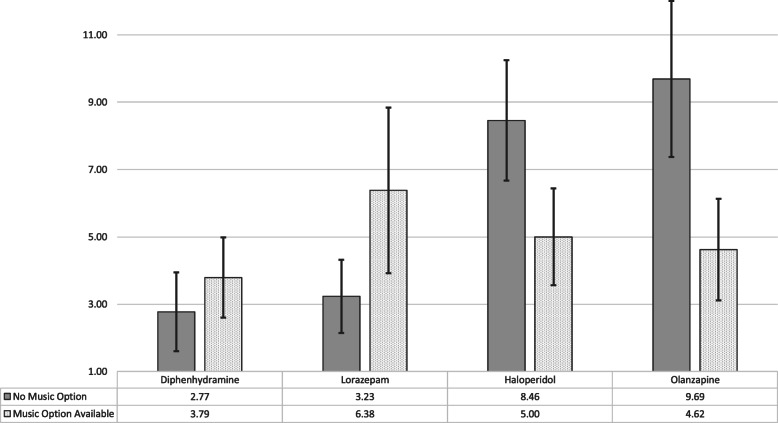
Change in Average Weekly PRN Administrations with 95% Confidence Interval

**Fig. 2:**
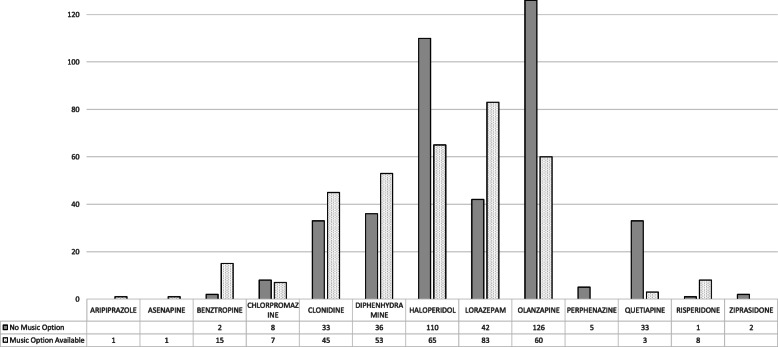
Absolute Number of Medication Administrations During Each Three-Month Period

PRN medications administered for agitation or anxiety were quantified through querying the electronic medical record. The indications for administering the PRN medications were documented by nursing staff, following approval by a medical physician. Electronic charts were reviewed retrospectively, to analyze the data collected over the preceding six-month period. Weekly average administrations, and total administrations for the 3 months with and without music, were calculated for each medication (lorazepam, olanzapine, etc.). To assess significance, the mean, standard deviation, standard error of the mean, and the 95% confidence intervals were calculated for the weekly PRN administrations for both three-month intervals. Bar graphs were created to visualize the data (Figs. 1 and 2).

Nursing staff were provided with categorical surveys regarding ease of implementation and effect on the unit milieu and asked to rate the perceived effectiveness of the intervention (Additional file 2). After completion of the study, surveys were collected, reviewed, and then categorized into different response groups ranging from positive to negative.

We had also intended to quantitatively score aggressive behavior, before and after music listening, using the Overt Agitation Severity Scale (OASS, published by Yudofsky et al., Additional file 3); however, due to limitations (identified in the “Project Limitations” section), this assessment method was discontinued shortly after the project’s onset. Instead, a Likert scale survey was implemented to subjectively assess patients’ response to music listening, prior to discharge from the inpatient unit.

### Inclusion criteria and participant characteristics

Any patient, 18 years or older, admitted to our acute psychiatric inpatient unit, who wished to have the music option available, was included in the project. Patients who did not want to listen to music or be included in the project were excluded. SUNY Upstate Medical University’s IRB determined the project does not meet the definition of human subject research under the purview of the IRB, according to federal regulations.

Data was collected between 5/18/2018 and 11/13/2018. The average daily census on the unit was 17 patients. Over the course of 6 months, 172 individuals, ranging in age from 18 to 82 years old (average 41 years old) participated in the project; 53.5% were male and 46.5% were female. Most patients were diagnosed with either a depressive, schizophrenia-spectrum, or trauma/stressor-related illness, often with substance-use comorbidity.

## Results

### PRN medication administration

The average weekly PRN medication administrations decreased significantly during the 3 months with music available, for both haloperidol (8.46 [+/− 1.79, *p* < 0.05] to 5.00 [+/− 1.44, *p* < 0.05] administrations/week) and olanzapine (9.69 [+/− 2.32, *p* < 0.05] to 4.62 [+/− 1.51, *p* < 0.05] administrations/week), compared to the 3 months prior to music implementation (Fig. 1). There was also an insignificant decrease in the use of chlorpromazine, quetiapine, perphenazine, and ziprasidone (Fig. 2). There was an increase in administrations of lorazepam, although insignificant (3.23 [+/− 1.09, *p* < 0.05] to 6.38 [+/− 2.46, *p* < 0.05] average administrations/week) (Fig. 1).

Overall, the total number of PRNs administered for anxiety and agitation (Fig. 2) decreased from 398 (prior to music availability) to 341 (while music was available), and the average weekly administrations decreased from 30.62 to 26.23, respectively. The most used medications for agitation included diphenhydramine, lorazepam, haloperidol, and olanzapine in ascending order.

### Patient and staff feedback

The patient survey responses were 96% positive (non-neutral; either agree or strongly agree with calming effect among the 31 surveys collected). There are multiple potential reasons for the low response rate: Patients would not complete the survey when they had to attend a group therapy session, needed to obtain medications due to worsening agitation despite music availability, or returned to sleep/relax in their rooms with refusal or early termination (less than 30 min). Anecdotally, patients tended to choose the same music genre on successive listening sessions, with Rap and Hip Hop being the most popular, per nursing staff. Additionally, many patients were initially ambivalent about using music for agitation, in the context of late evening arrival, withdrawal symptoms or untreated psychiatric symptoms, but later opted to use music for de-escalation.

Nurses agreed that the project was easy to implement; 56% (among nine surveys collected) agreed that music helped to calm patients down (Table 1). Staff were initially concerned regarding swallow risk, especially if the headphones were broken into smaller pieces. Fortunately, no notable damage occurred to the headphones and they remained in use for the duration of the study without need for replacement. There were no attempts to swallow or use the headphones for self-injury.

**Table 1:** Responses to Nursing Survey Questions

Nursing Survey	Strongly Disagree	Disagree	Undecided	Agree	Strongly Agree
Easy to Implement	0	4	0	5	0
Quieter Milieu	2	4	1	2	0
Effectively Calmed Patient	3	1	0	5	0
Total	5	9	1	12	0

#### Other exploratory findings

The mean length of hospital stay decreased by 2.29 days (10.49 to 8.20 days) and the median length of stay decreased by 1.5 days (6.5 to 5.0 days) during the 3 months when music was available compared to baseline. Assaults decreased from three to zero and incidents of property damage decreased from two to one. Seclusion events also decreased from 57 to 25. Seclusions were utilized when a nursing staff member, in consultation with a psychiatric resident or attending physician, determined a patient was agitated or anxious and posed a threat of harm to themselves, staff members, or other patients. To ensure safety, patients were secluded when medications insufficiently controlled their high-risk behaviors.

## Discussion

In this quality improvement project, we set out to study the effectiveness and feasibility of using music as an adjunct or replacement for pharmacologic agitation management on an inpatient psychiatric unit. The project was well-received by most patients and staff members. The results suggest that music listening may help to reduce reliance on PRN agitation medications.

During the period when music was available for agitated patients, haloperidol and olanzapine administration significantly decreased (Fig. 1). Haloperidol and olanzapine are the most used PRN antipsychotic medications for agitation on our inpatient psychiatric unit. Conversely, diphenhydramine and lorazepam use increased, although non-significantly (Fig. 1). These PRN medications are also given for EPS and mild anxiety. Furthermore, lorazepam is given to patients in alcohol or benzodiazepine withdrawal and for seizure prophylaxis. In these cases, music therapy would not serve as a suitable substitute, due to the substantial health risk.

In addition to excluding administrations for EPS, withdrawal, and seizures, we also attempted to exclude doses provided for treating psychotic or acute manic symptoms (e.g. auditory hallucinations). The PRN administrations were not subdivided by patient admission diagnosis, due to most patients having multiple overlapping diagnoses with waxing and waning symptoms during their stay. Therefore, only the primary acute indication at the time of administration was considered. The rough distribution of admission diagnoses was consistent in both the pre- and post-music availability periods.

Though underpowered, we observed an overall reduction of seclusions, assaults, and property damage between the two study periods. Music utilization may have prevented some patients from traumatic chemical and/or physical involuntary restraint. Ideally, opting for music allowed patients to maintain a sense of agency, while trying to address their unmet needs.

The average length of stay in the inpatient psychiatric unit unexpectedly decreased by greater than 2 days, between the two observation periods. This is only a preliminary finding and offered as an exploratory outcome for future studies, as a decrease in the length of psychiatric hospitalization could have significant therapeutic benefits. For example, patients diagnosed with borderline personality disorder may regress when hospitalized for extended periods of time [22, 23]. Reductions in length of stay also minimize the financial burden placed on patients with mental health diagnoses, a population that is significantly less likely to have access to health insurance [24].

### Project limitations

Prior to starting the project, we conducted meetings with our nursing team to obtain feedback regarding possible challenges of implementation. In addition, each staff member received printed instructions to explain the new music protocol. Despite these measures, only about one-third of unit staff completed the project and the final survey. 56% of completed surveys indicated that staff felt music helped to reduce agitation and 44% indicated that staff thought the project was difficult to implement.

The initial decision to utilize the OASS to quantitatively score agitation severity before and after music listening, likely contributed to the difficulty of implementation. To complete this scale, the frequency and intensity of 12 different agitation-associated behaviors (e.g., pacing, slapping, vocal perseveration) had to be recorded within a limited window of time. Nursing staff were unable to consistently complete this scale, so its use was discontinued shortly after project onset. Instead, Likert scales were implemented to gauge patient and staff satisfaction with the intervention. Another factor likely hindering implementation, involved the difficulty of monitoring patients enrolled in the project, while simultaneously attending to the needs of other patients on the unit. As there are typically four to five nurses working per shift and 15 to 21 patients hospitalized on the unit at a given time, each nurse is responsible for three to five patients. Finally, though some agitated patients expressed interest in participating in the project, their previous patterns of violence or hostility may have fostered reluctance among nursing staff to offer music. Half of the surveys completed by nursing staff revealed that they were concerned that patients would either damage or misplace headphones, exhibit increased intrusive behavior at the nursing station, or resist returning the headphones. Music was only provided to those patients who requested it. Therefore, we cannot draw conclusions regarding the consequences of providing music to agitated patients who did not express interest. Medication adjustments routinely occur on our inpatient unit, secondary to side effects or lack of perceived benefit. Our study did not include analysis of standing medication changes, which serves as an additional confounding variable in interpreting results.

Future studies should consider accounting for differences in scheduled medications between study groups. Additionally, if researchers seek to expand upon this project’s findings, certain aspects of its protocol could be altered to ease implementation. Because the OASS was discontinued early in the project in favor of the more straightforward Likert scales, more simplistic scales could be used in future projects at their onset, to reduce the burden on unit staff. Ideally, select unit staff could be freed of other clinical responsibilities, so that they may allocate time to ensure accurate implementation of the music intervention protocol. Alternatively, a trained clinical researcher could be employed, though this would likely incur additional costs.

## Conclusion

Our project suggests that music listening may play a significant role in reducing the utilization of PRN agitation medications on acute inpatient psychiatric units. We observed a significant decrease in the administration of PRN olanzapine and haloperidol during the period when music was available, but more studies are needed to control for confounding variables, such as underlying diagnosis and circumstances preceding hospitalization. Due to the overall positive reception and ease of implementation, we continue to employ music as a means of emotion regulation on SUNY Upstate University Hospital’s adult inpatient psychiatric unit. The project has been low-cost, well received by staff and patients, and neither equipment damage nor swallowing incidents have occurred. Patients continue to request music as a coping strategy for agitation, demonstrating the feasibility of this project.

Future studies may expand on these findings by analyzing the frequency of medication administration among patients of varying diagnostic categories (e.g., Schizophrenia Spectrum and Other Psychotic Disorders, Personality Disorders, Substance-Related and Addictive Disorders). In addition to the exploratory outcomes already mentioned, other potential areas of focus include number of readmissions, rate of participation in inpatient psychotherapy groups, and overall medication compliance. Though our project emphasized maximizing autonomy by allowing patients to choose a preferred music genre, future studies might consider the relationship between the selected genre of music (e.g., Classical, Rock, Jazz), and the degree of agitation reduction. Furthermore, as there is substantial variation within musical genres, future research could also examine the effect of intrinsic musical properties (e.g., timbre and cadence) on patient agitation levels.

## Supplementary Information

**Additional file 1.**

**Additional file 2.**

**Additional file 3.**

## Data Availability

The datasets obtained and/or analyzed during the current study are available from the corresponding author on reasonable request.

## Abbreviations

ACC: Anterior cingulate cortex
BETA: Best practices in evaluation and treatment of agitation
EPS: Extrapyramidal symptoms
PRN: As-needed
IRB: Institutional review board
OASS: Overt agitation severity scale
OFC: Orbitofrontal cortex
