# Supplementary material for: Mindful Melody: feasibility of implementing music listening on an inpatient psychiatric unit and its relation to the use of as needed medications for acute agitation
Source: BMC Psychiatry. 2021 Mar 6;21:132. doi: 10.1186/s12888-021-03127-z (PMC7937203; doi:10.1186/s12888-021-03127-z)
Supplement: Supplementary file 1 — Additional file 1. [file 12888_2021_3127_MOESM1_ESM.pdf]

Additional file 1

Name___________________________________

MUSIC FOR AGITATION PROJECT

Patient's Feedback Form

Please give to the patient at the end of 30 or less minutes listening to the music.

Music helped me to calm down:

Strongly Disagree	Disagree	Undecided	Agree	Strongly Agree
