# Supplementary material for: Mindful Melody: feasibility of implementing music listening on an inpatient psychiatric unit and its relation to the use of as needed medications for acute agitation
Source: BMC Psychiatry. 2021 Mar 6;21:132. doi: 10.1186/s12888-021-03127-z (PMC7937203; doi:10.1186/s12888-021-03127-z)
Supplement: Supplementary file 2 — Additional file 2. [file 12888_2021_3127_MOESM2_ESM.pdf]

Additional file 2

MUSIC FOR AGITATION PROJECT

Staff Feedback Form

Please complete at the end of your shift.

1. The project was relatively easy to implement:

Strongly Disagree	Disagree	Undecided	Agree	Strongly Agree

2. The project helped the unit milieu to be more quiet:

Strongly Disagree	Disagree	Undecided	Agree	Strongly Agree

3. The project helped the patient to calm down:

Strongly Disagree	Disagree	Undecided	Agree	Strongly Agree

4. Comments:
