# Supplementary material for: Mindful Melody: feasibility of implementing music listening on an inpatient psychiatric unit and its relation to the use of as needed medications for acute agitation
Source: BMC Psychiatry. 2021 Mar 6;21:132. doi: 10.1186/s12888-021-03127-z (PMC7937203; doi:10.1186/s12888-021-03127-z)
Supplement: Supplementary file 3 — Additional file 3. [file 12888_2021_3127_MOESM3_ESM.pdf]

Additional file 3

Patient Name: ______________________ Name of Rater: ______________________

Time of Observation: _________ AM / PM Date of Observation: _________

NURSING STAFF: *For each behavior, circle the corresponding frequency after 15 minutes of observation.*

Overt Agitation Severity Scale

Yudofsky SC, Kopecky HJ, Kunik M, Silver JM, Endicott J

**INTENSITY (I)**	**BEHAVIOR**	**FREQUENCY (F)**					**SEVERITY SCORE (SS)**
**A.**	**Vocalizations & Oral/Facial Movements**	**Not Present**	**Rarely**	**Some of the time**	**Most of the time**	**Always present**	**RESEARCH STAFF ONLY (IxF=SS)**
1	Whimpering, whining, moaning, grunting, crying	0	1	2	3	4	
2	Smacking or licking of lips, chewing, clenching jaw; licking, grimacing, spitting	0	1	2	3	4	
3	Rocking, twisting, banging of head	0	1	2	3	4	
4	Vocal perseverating, screaming, cursing, threatening, wailing	0	1	2	3	4	
**B.**	**Upper Torso & Upper Extremity Movements**						
1	Tapping fingers, fidgeting, or wringing of hands, swinging or flailing arms	0	1	2	3	4	
2	Task perseverating (e.g., opening and closing drawers, folding and unfolding clothes, picking at objects, clothes, or self, pulling at own hair)	0	1	2	3	4	
3	Rocking (back & forth), bobbing (up and down), twisting, writhing of torso; rubbing or masturbating self	0	1	2	3	4	
4	Slapping, swatting, hitting at objects or others	0	1	2	3	4	
**C.**	**Lower Extremity Movements**						
1	Tapping toes, clenching toes, tapping heel, extending, flexing or twisting foot	0	1	2	3	4	
2	Shaking legs, tapping knees and/or thighs, thrusting pelvis, stomping	0	1	2	3	4	
3	Pacing, wandering	0	1	2	3	4	
4	Thrashing legs, kicking at objects or others	0	1	2	3	4	

RESEARCH STAFF: *For each behavior exhibited, calculate the Severity Score (IxF) and record the sum as the total Overt Agitation Severity Score (OASS). Subtract the baseline OASS to account for consistently present neuromuscular symptoms (e.g. tardive dyskinesia) and create a revised (final) OASS.*

Total: OASS

Baseline: OASS

Revised: OASS
